# *Glyma11g13220*, a homolog of the vernalization pathway gene *VERNALIZATION 1* from soybean [*Glycine max* (L.) *Merr.*], promotes flowering in *Arabidopsis thaliana*

**DOI:** 10.1186/s12870-015-0602-6

**Published:** 2015-09-29

**Authors:** Jing Lü, Haicui Suo, Rong Yi, Qibin Ma, Hai Nian

**Affiliations:** The State Key Laboratory for Conservation and Utilization of Subtropical Agro-Bioresources, South China Agricultural University, Guangzhou, China; The Key Laboratory of Plant Molecular Breeding, South China Agricultural University, Guangzhou, China; The Guangdong Subcenter of the National Center for Soybean Improvement, College of Agriculture, South China Agricultural University, Guangzhou, China; The Crop Research Institute, Guangdong Academy of Agricultural Sciences, Guangzhou, China; Guangdong Provincial Key Laboratory of Crop Genetics and Improvement, Guangzhou, China

## Abstract

**Background:**

The precise timing of flowering is fundamental to successful reproduction, and has dramatic significance for crop yields. Although prolonged low temperatures are not required for flowering induction in soybean, vernalization pathway genes have been retained during the evolution of this species. Little information is currently available in regarding these genes in soybean.

**Results:**

We were able to detect the expression of *Glyma11g13220* in different organs at all monitored developmental stages in soybean. *Glyma11g13220* expression was higher in leaves and pods than in shoot apexes and stems. In addition, *Glyma11g13220* was responsive to photoperiod and low temperature in soybean. Furthermore, Glyma11g13220 was found to be a nuclear-localized protein. Over-expression of *Glyma11g13220* in an Arabidopsis Columbia-0 (Col-0) background resulted in early flowering. Quantitative real-time PCR analysis revealed that transcript levels of flower repressor *FLOWERING LOCUS C* (*FLC*), and *FD* decreased significantly in transgenic Arabidopsis compared with wild-type Col-0, while the expression of *VERNALIZATION INSENSITIVE 3* (*VIN3*) and *FLOWERING LOCUS T* (*FT*) noticeably increased.

**Conclusions:**

Our results suggest that Glyma11g13220, a homolog of Arabidopsis VRN1, is a functional protein. *Glyma11g13220*, which is responsive to photoperiod and low temperature in soybean, may participate in the vernalization pathway in Arabidopsis and help regulate flowering time. Arabidopsis *VRN1* and *Glyma11g13220* exhibit conserved as well as diverged functions.

**Electronic supplementary material:**

The online version of this article (doi:10.1186/s12870-015-0602-6) contains supplementary material, which is available to authorized users

## Background

Flowering, which refers to the transition from the vegetative to the reproductive phase, is one of the most crucial events in the plant life cycle. The precise timing of flowering is controlled by external environmental cues and endogenous developmental signals. Correct timing is fundamental to successful reproduction and has dramatic significance for crop yields [1]. Five genetic pathways relevant to flowering have been identified in the model species *Arabidopsis thaliana*, namely, photoperiod, vernalization, gibberellic acid, autonomous and aging pathways [2]. Photoperiod and vernalization pathways regulate flowering time by perceiving environmental changes, such as alterations in day length in the case of the former and prolonged low temperature in the latter. In contrast, gibberellic acid, autonomous and aging pathways responses to flowering are internally controlled [2]. Nevertheless, increasing evidence is revealing that the genetically defined pathways that regulate flowering time are connected. For example, these pathways are integrated by a series of downstream flowering integrator genes, including *FLOWERING LOCUS T* (*FT*) and *SUPPRESSOR OF CONSTANS 1* (*SOC1*), whose outputs are subsequently conveyed to floral meristem identity genes, such as *APETALA 1* (*AP1*) and *LEAFY* (*LFY*), that trigger flowering [3].

Flowering integrators are regulated in two completely opposite ways by two central upstream genes: *CONSTANS* (*CO*) and *FLOWERING LOCUS C* (*FLC*) [4, 5]. One of the integrators, *FT*, is controlled by both *CO* and *FLC* [4, 6]. *CO*, a core component of the photoperiod pathway, encodes a zinc finger protein, acts as a floral activator and is mediated by the circadian clock [7]. *FLC*, in contrast, encodes a MADS-box transcription factor that acts as a repressor of flowering [6]. At present, many pathways have been reported to regulate *FLC* via different chromatin pathways and co-transcriptional mechanisms involving cold-induced long antisense intragenic RNA (COOLAIR) transcripts [8, 9]. One of these pathways is the autonomous pathway in which alternative processing of COOLAIR transcripts leads to gene body histone K4 demethylation and *FLC* down-regulation [9]. In another such pathway, the vernalization pathway, prolonged cold elevates COOLAIR transcription and silences *FLC* in a Polycomb-mediated epigenetic process [10, 11].

Vernalization is the process promoting flowering in plants after prolonged low temperature treatment (1 to 3 months at about 4 °C) [12]. In Arabidopsis, the molecular mechanism of vernalization has been studied by identifying the functions of a set of *VRN* genes. *VRN1* encodes a plant-specific protein that binds DNA in a non–sequence-specific manner *in vitro* [13]. The VRN1 protein sequence possesses two B3 DNA-binding domains that were first discovered in the maize protein VIVIPAROUS1 (VP1) [14] as well as two putative PEST protein-turnover domains [15] and a nuclear localization signal sequence [13]. Although over-expression of *VRN1* causes early flowering in Arabidopsis, *vrn1* mutants of Arabidopsis do not delay flowering time—they merely reduce vernalization response [13]. Briefly, *VRN1* regulates flowering time by stably repressing the floral repressor *FLC* [13]. *VRN1* is also involved in other processes essential for Arabidopsis development [16]. Other *VRN* genes participating in regulation of flowering time through the vernalization pathway have also been identified. *VERNALIZATION 2* (*VRN2*), which encodes a nuclear-localized zinc finger, is a homolog of the *Drosophila* Polycomb protein SU(Z)12. Both *VRN1* and *VRN2* maintain the repression of *FLC* epigenetically [17]. *VERNALIZATION 3* (*VIN3*), encoding a plant homeodomain finger protein, is only expressed during vernalization and represses *FLC* [18]. Compared with *VRN1* and *VRN2*, which maintain *FLC* silencing, *VIN3* is essential for establishing *FLC* repression during vernalization [19]. *VERNALIZATION 5* (*VRN5*), a *VIN3*-related protein, is constitutively expressed [20, 21].

Soybean [*Glycine max* (L.) *Merr.*], a typically photoperiod-sensitive plant, is classified as a short-day species. Because of this photoperiod sensitivity, soybean cultivation has long been limited to a very narrow latitudinal range. The recent availability of the soybean draft genome sequence has accelerated the study of soybean flowering. Comparative genomic analysis of soybean and Arabidopsis flowering genes has revealed similar flowering pathways in these two species [22, 23]. Interestingly, vernalization pathway genes are also found in soybean, which does not need to undergo a prolonged low temperature treatment before flowering [22]. In our preliminary research, Arabidopsis *DREB1A* driven by the 35S promoter was introduced into soybean, yielding transgenic plants that displayed delayed flowering [24]. An expression analysis of flowering time showed that the vernalization pathway gene, Glyma11g13220 was strongly up-regulated in the transgenic plants (unpublished results). We thus speculate that this gene may play important roles in the regulation of flowering time. In the study reported here, the functions of *Glyma11g13220*, a homolog of Arabidopsis *VRN1*, were investigated for the first time. We found that *Glyma11g13220* was responsive to photoperiod and low temperature in soybean and that heterologous expression of *Glyma11g13220* in Arabidopsis Columbia-0 (Col-0) caused early flowering. In transgenic Arabidopsis, the expressions of *FD* and flower repressor *FLC* obviously decreased and the expressions of *VIN3* and floral integrator *FT* increased significantly. These results imply that Glyma11g13220 is a functional protein similar to VRN1 in Arabidopsis and may play a pivotal role in regulating flowering time through the vernalization pathway.

## Results

### Isolation and sequence analysis of *Glyma11g13220*

**Fig. 1:**
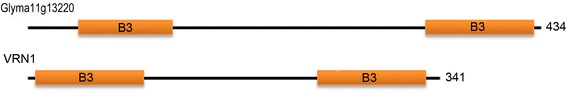
Diagram of Glyma11g13220 and Arabidopsis VRN1 domain organization. The two B3 domains of Glyma11g13220 are located between amino acids 40–120 and 334–429, while those of Arabidopsis VRN1 are positioned between amino acids 5–96 and 244–332

**Fig. 2:**
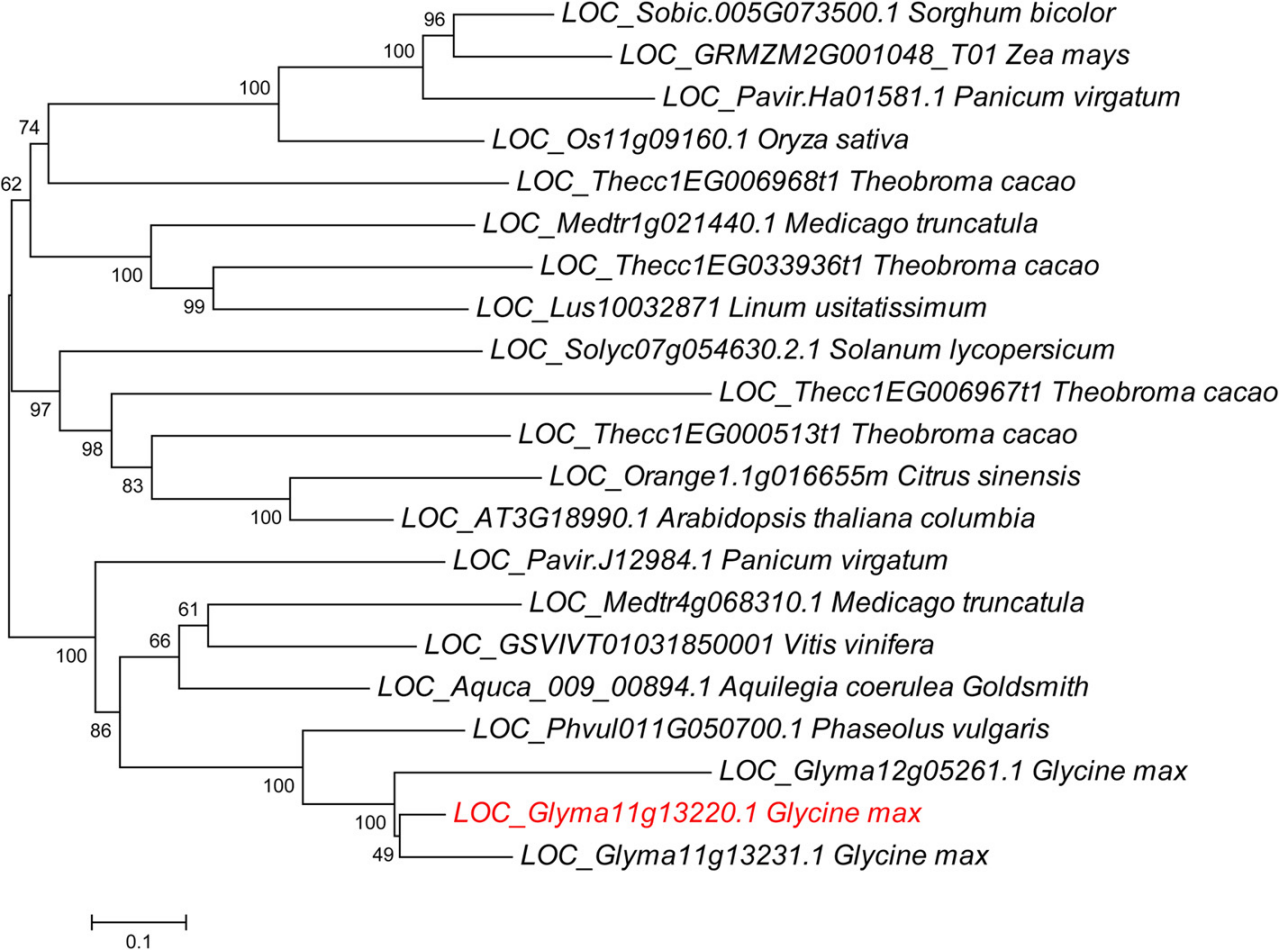
Phylogenetic tree of Glyma11g13220 and related proteins. To identify homologs, the Glyma11g13220 protein sequence was used as the query in BlastP searches. Multiple sequence alignment of protein sequences was carried out using Clustal Omega. The phylogenetic tree was constructed using the aligned sequences according to the neighbor-joining algorithm as implemented in MEGA 5.0 with 1,000 bootstrap replicates

As inferred from previous results in our laboratory involving *AtDREB1A*-overexpressing soybean plants exhibiting delaying flowering [24], Glyma11g13220.1 may play important roles in flowering time regulation (unpublished results). Sequence information for the flowering-induced gene Glyma11g13220.1 was obtained from the Phytozome v.9.1 database [25]. Although VRN1 was not the Arabidopsis B3 protein having the highest similarity to Glyma11g13220 (Additional file 1), *Glyma11g13220.1* was predict to be a homolog of Arabidopsis *VRN1* in accordance with previous comparative genomic analyses of soybean flowering genes [22, 26]. To further characterize the function of *Glyma11g13220* in regulation of flowering time, we isolated the gene from the soybean cultivar Huachun5. The *Glyma11g13220* sequence was 1,863 bp long and contained a 175-bp 5′ untranslated region (UTR), a 383-bp 3′ UTR and a 1,305-bp open reading frame. BLAST analysis indicated that this sequence was consistent with the William 82 soybean reference sequence. Glyma11g13220 was predicted to encode a protein of 434 amino acids. Two putative B3 DNA domains were also separately identified at amino acid residues 40–120 and 334–429 (Fig. 1). Phylogenetic analysis revealed that related homologs of *Glyma11g13220* were mainly found in monocots and especially in leguminous plants, but not in lower plants, animals or microbes. This distribution pattern indicates that this type of gene is specific to higher plants (Fig. 2). Even though Glyma11g13220 shared only weak amino acid sequence identity with VRN1 in Arabidopsis (Additional file 2), both of these genes had two conserved B3 DNA domains (Fig. 1). The presence of these shared domains suggests that the function of *Glyma11g13220* may be similar to that of Arabidopsis *VRN1*.

### Sequence analysis of the *Glyma11g13220* promoter

In an attempt to elucidate the possible factors associated with the regulation of *Glyma11g13220* expression, we analyzed the promoter region using the PLANTCARE database [27] and found several putative *cis*-elements. All of the identified *cis*-elements are listed in Table 1. The elements in this region included light-responsive elements (3-AF1, ACE, AT1, G-BOX, GT-1 and LAMP), abiotic stress-responsive elements (MBS, DRE, TC-rich and HSE), and plant hormone-related flowering elements (GARE, ABRE and TCA). The presence of many different potential *cis*-elements in the upstream region of *Glyma11g13220* suggests that the gene is regulated by multiple external environmental and internal hormonal cues and especially by light conditions.

**Table 1:** Putative *cis*-elements in the *Glyma11g13220* promoter

*cis*-element	Position (From ATG)	Sequence (5′-3′)
Light regulation elements		
3-AF1 binding site	−1444(+)	ATGAGATATTT
ACE	−1387(−)	CAAACGTATT
AT1-motif	−511(+)	AATTATTATTTATT
Box 4	−126(+),−560(+),−615(+),−752(+),−1183(+)	ATTAAT
Box I	−390(+),−1363(+)	TTTCAAA
G-Box	−156(+)	TACGTG
I-box	−841(−)	GTAAAAGGCC
LAMP-element	−63(+)	CTTTATCA
chs-CMA1a	−1452(−)	TTACTTAA
Tissue-specific and development-related elements		
GCN4_motif	−1023(+)	TGAGTCA
Skn-1_motif	−230(+),-1020(+),−1111(+)	GTCAT
Circadian	−1220(+)	AAAAGATATC
GARE-motif	−880(−)	AAACAGA
TCA	−199(−)	GAGAATAATA
ABRE	−156(+)	TACGTG
Abiotic stress response elements		
MBS	−1081(−),−649(+)	(C/T)AACTG
HSE	−1307(−),−628(+)	A(A/G)AAAATTT(A/G)
DRE	−170(+)	TACCGACAT
TC-rich repeats	−1230(+)	ATTTTCTTAA

### Transcript profiling of *Glyma11g13220* in soybean

**Fig. 3:**
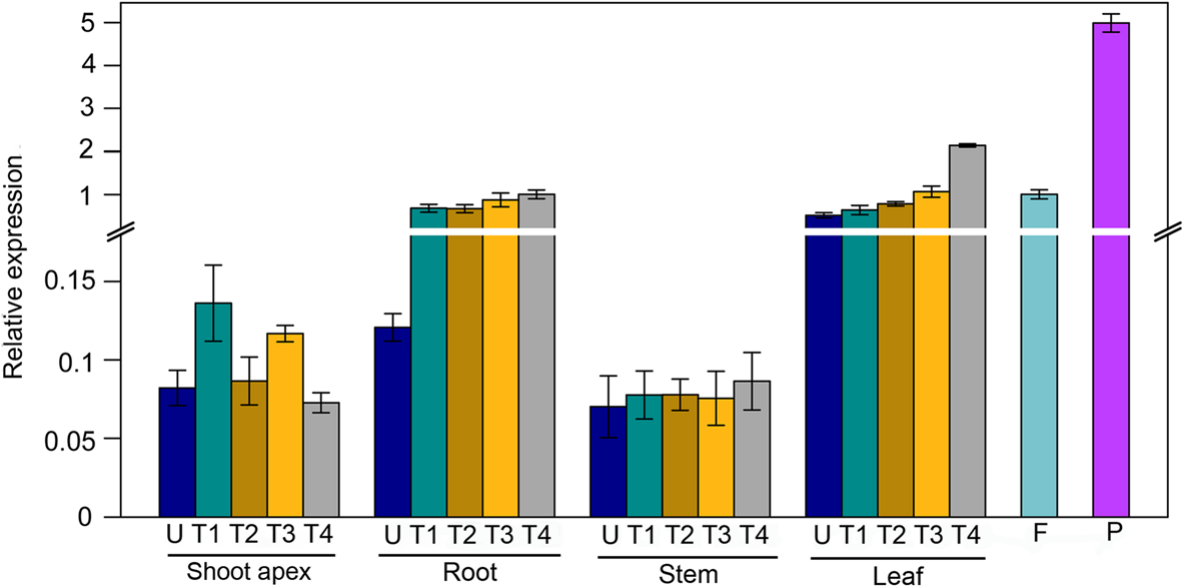
Transcript profiling of *Glyma11g13220* in soybean based on quantitative real-time PCR analysis of *Glyma11g13220* in different organs at different developmental stages under short-day conditions. U, untrifoliate period; T1, first trifoliate period; T2, second trifoliate period; T3, third trifoliate period; T4, fourth trifoliate period; Shoot apex (including apical meristem and immature leaves); F, flower; P, pod 14 days after flowering. Expression levels are normalized to *Gm*β*-tubulin* (Glyma20g27280). Values are means ± SD of three biological replicates, with each measurement repeated three times

To study the underlying role of *Glyma11g13220* in flowering during the soybean development process, we used quantitative real-time PCR (qRT-PCR) to analyze its transcription levels in multiple organs, including leaves, stems, roots, shoot apexes, flowers and pods, at different vegetative and reproductive growth stages under short-day conditions (Fig. 3). *Glyma11g13220* expression was readily detected in all organs at all monitored developmental stages. *Glyma11g13220* transcript levels were higher in leaves and pods than in other analyzed organs. *Glyma11g13220* expression levels gradually increased in leaves during the development period, reaching their maximum before flowering. In contrast, expression was very low in shoot apexes and stems. This observed pattern suggests that *Glyma11g13220* plays a role prior to flowering.

### Expression patterns of *Glyma11g13220* in response to different light conditions

**Fig. 4:**
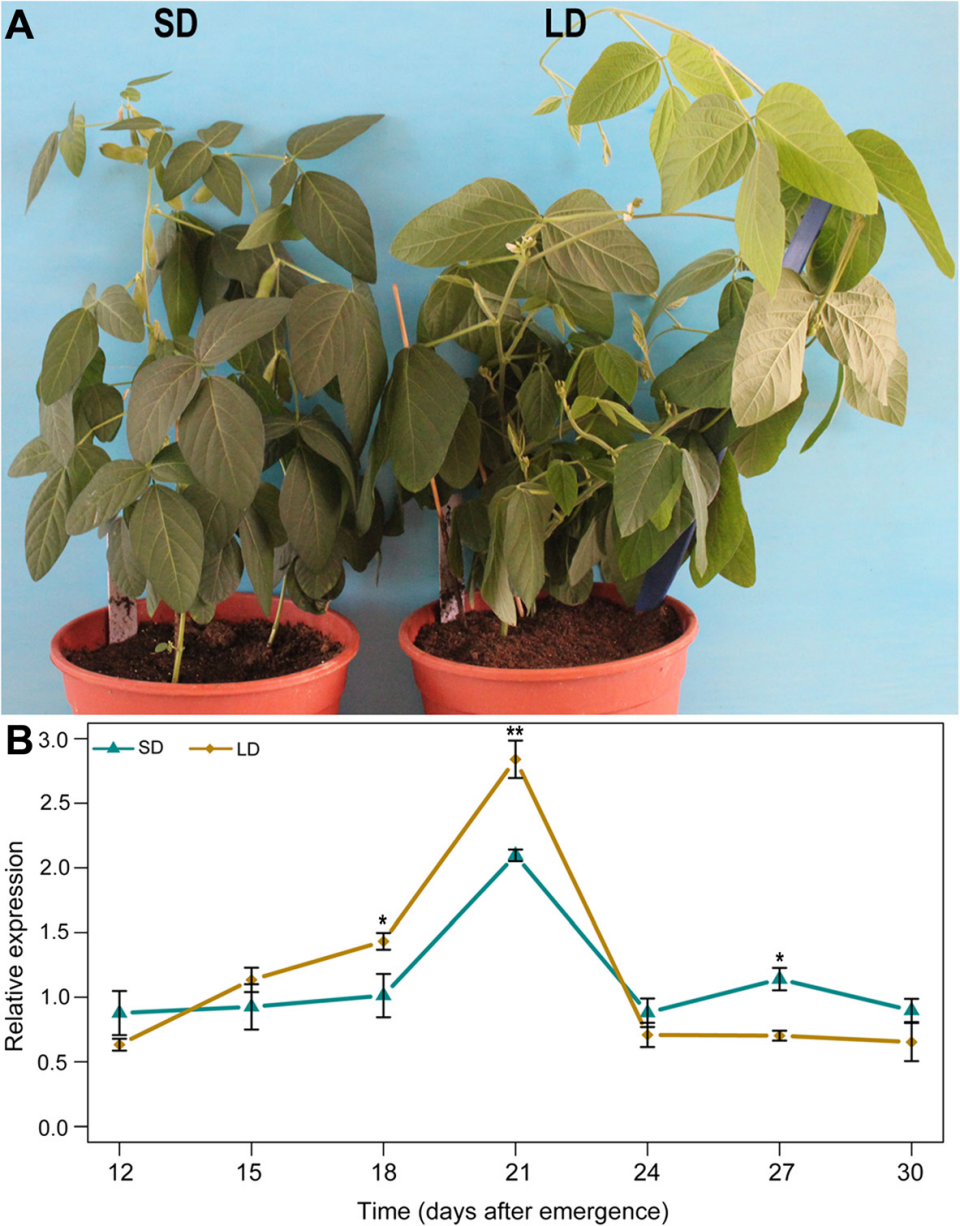
Expression patterns of *Glyma11g13220* under different light conditions. All seedlings were grown under short-day conditions until 10 days after emergence (DAE), at which point half of the seedlings were transferred to long-day conditions. Fully expanded trifoliate leaves were sampled from three individual plants growing under short- and long-day conditions 12 h after dawn at 12, 15, 18, 21, 24, 27 and 30 DAE. **a** Image obtained approximately 53 DAE (SD, short-day conditions; LD, long-day conditions). **b** Quantitative real-time PCR analysis of *Glyma11g13220* under short- and long-day conditions at 12, 15, 18, 21, 24, 27 and 30 DAE. Expression levels are normalized to *Gm*β*-tubulin* (Glyma20g27280). Values are means ± SD of three biological replicates, with each measurement repeated three times. Significant differences based on the *t*-test are denoted by asterisks: * *p* < 0.05, ** *p* < 0.01

Because we found many light-responsive *cis*-elements in the *Glyma11g13220* promoter (Table 1), we investigated whether *Glyma11g13220* is photoperiod responsive. To examine the photoperiod sensitivity of this gene, we observed the phenotype of Huachun5 and analyzed the time course-dependent expression patterns of *Glyma11g13220* in soybean under both short- and long-day conditions. As can be seen in Fig. 4a and Additional file 3, Huachun5 plants flowered significantly earlier under short-day conditions than under long-day ones. Approximately 53 days after emergence (DAE), soybean plants grown under short-day conditions were in the full of pods period, whereas plants under long-day conditions were still in the initial flowering period. This phenotypic difference demonstrates that Huachun5 is sensitive to photoperiod. With respect to *Glyma11g13220* expression over time, transcript levels remained unchanged during the initial period under short-day conditions; they subsequently increased sharply to a maximum at 21 DAE and then decreased. Under long-day conditions, in contrast, *Glyma11g13220* expression was gradually up-regulated, showing a peak at 21 DAE with reduced expression thereafter. At 18, 21 and 27 DAE, *Glyma11g13220* expression existed significantly different between under short- and long-day conditions. This result implies that *Glyma11g13220* is photoperiod responsive in soybean.

### Subcellular localization of Glyma11g13220 protein

**Fig. 5:**
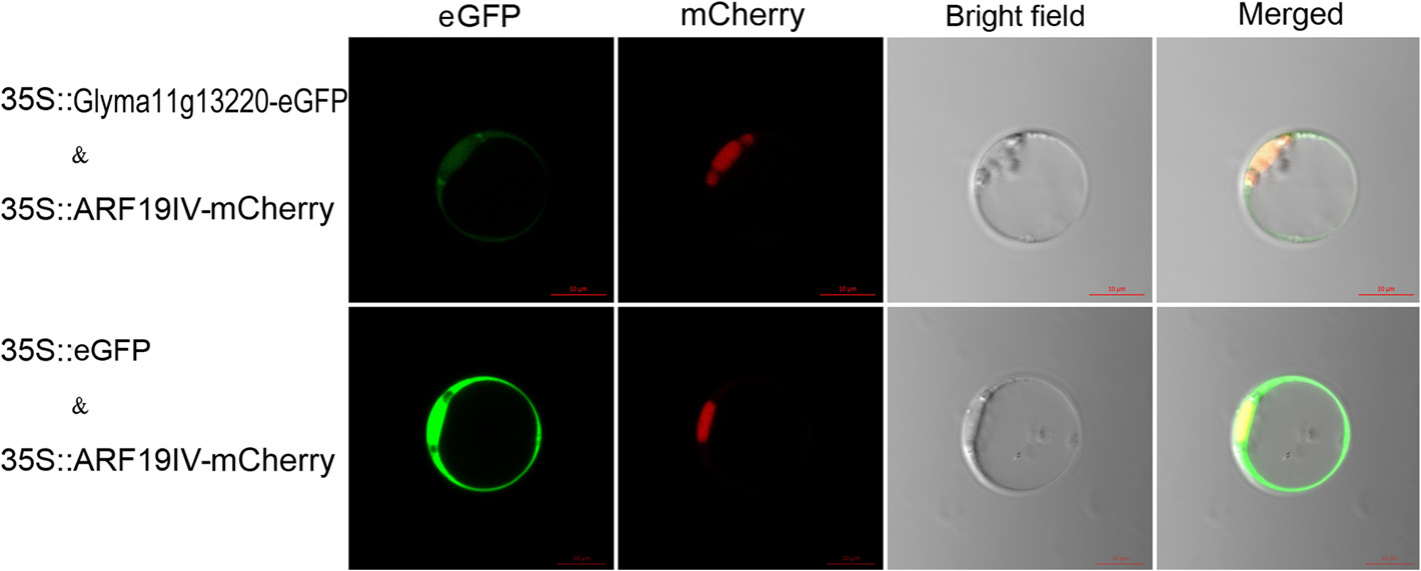
Subcellular localization of Glyma11g13220-GFP fusion protein. Constructs 35S::*Glyma11g13220-eGFP* and 35S::*eGFP* were separately co-transformed into rice protoplast cells with 35S::*ARF19IV-mCherry*. The cells were observed under a confocal laser microscope. ARF19IV-mCherry was used as a nuclear marker protein. Scale bars, 10 μm

To understand the potential function of *Glyma11g13220*, we examined the subcellular localization of Glyma11g13220 in rice protoplasts. As shown in Fig. 5, the enhanced green fluorescent protein (eGFP) fluorescence signal of Glyma11g13220 clearly overlapped with the mCherry fluorescence signal, whereas no obvious fluorescence signal was detected in the cytoplasm. Conversely, the eGFP fluorescence signal of the empty control was distributed throughout the whole cell. The results of this experiment indicate that Glyma11g13220 is mainly a nuclear-localized protein.

### Early flowering in Arabidopsis caused by ectopic expression of *Glyma11g13220*

**Fig. 6:**
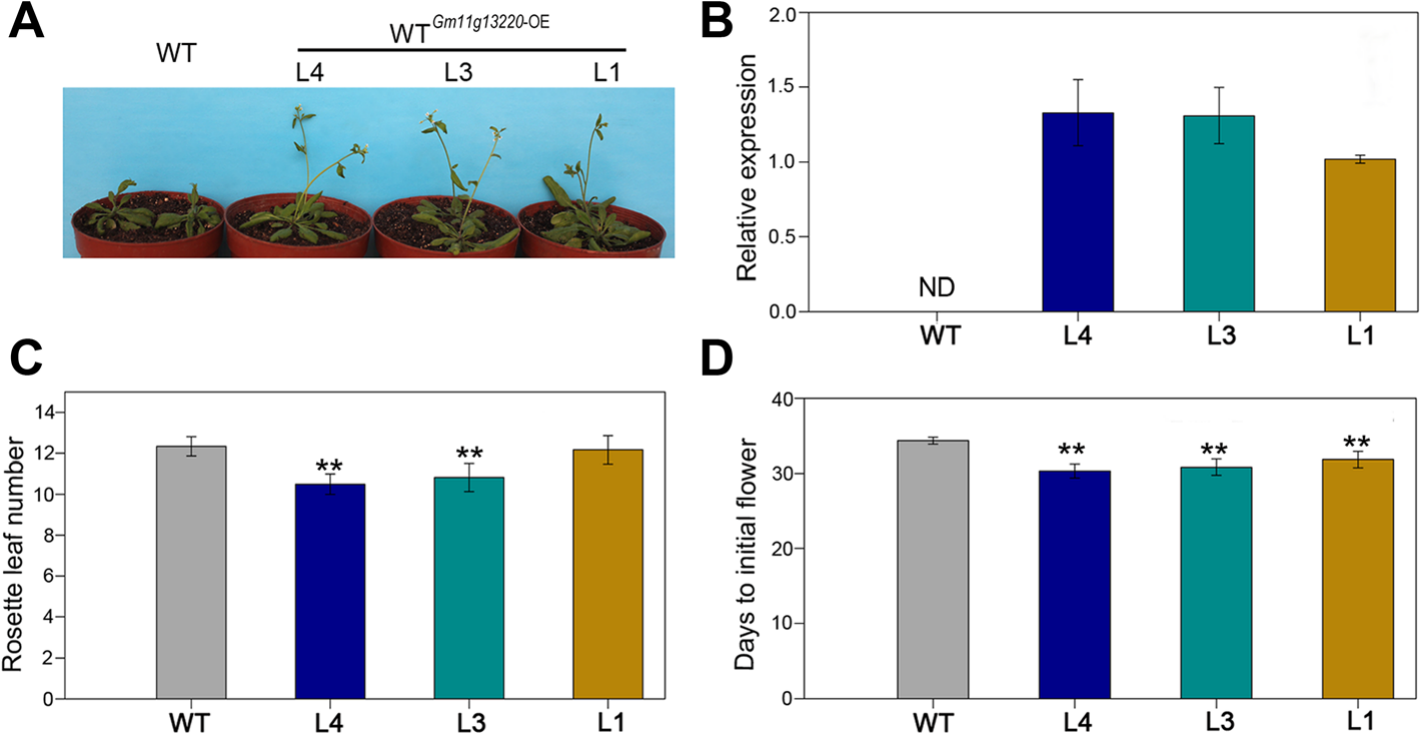
Heterologous expression of *Glyma11g13220* in Arabidopsis. **a** Phenotypic comparison between transgenic and wild-type (Col-0) plants. One-month-old plants were photographed. **b** Quantitative real-time PCR analysis of *Glyma11g13220* in transgenic plants. ND, not detected. Values are means ± SD of three biological replicates, with each measurement repeated three times. **c** Rosette leaf numbers of transgenic and wild-type (Col-0) plants during flowering. Values are means ± SD; *t*-test: * *p* < 0.05, ** *p* < 0.01. At least six plants were counted for each line. **d** Days until initial flowering of transgenic and wild-type (Col-0) plants. Values are means ± SD; *t*-test: * *p* < 0.05, ** *p* < 0.01. At least six plants were counted for each line

**Fig. 7:**
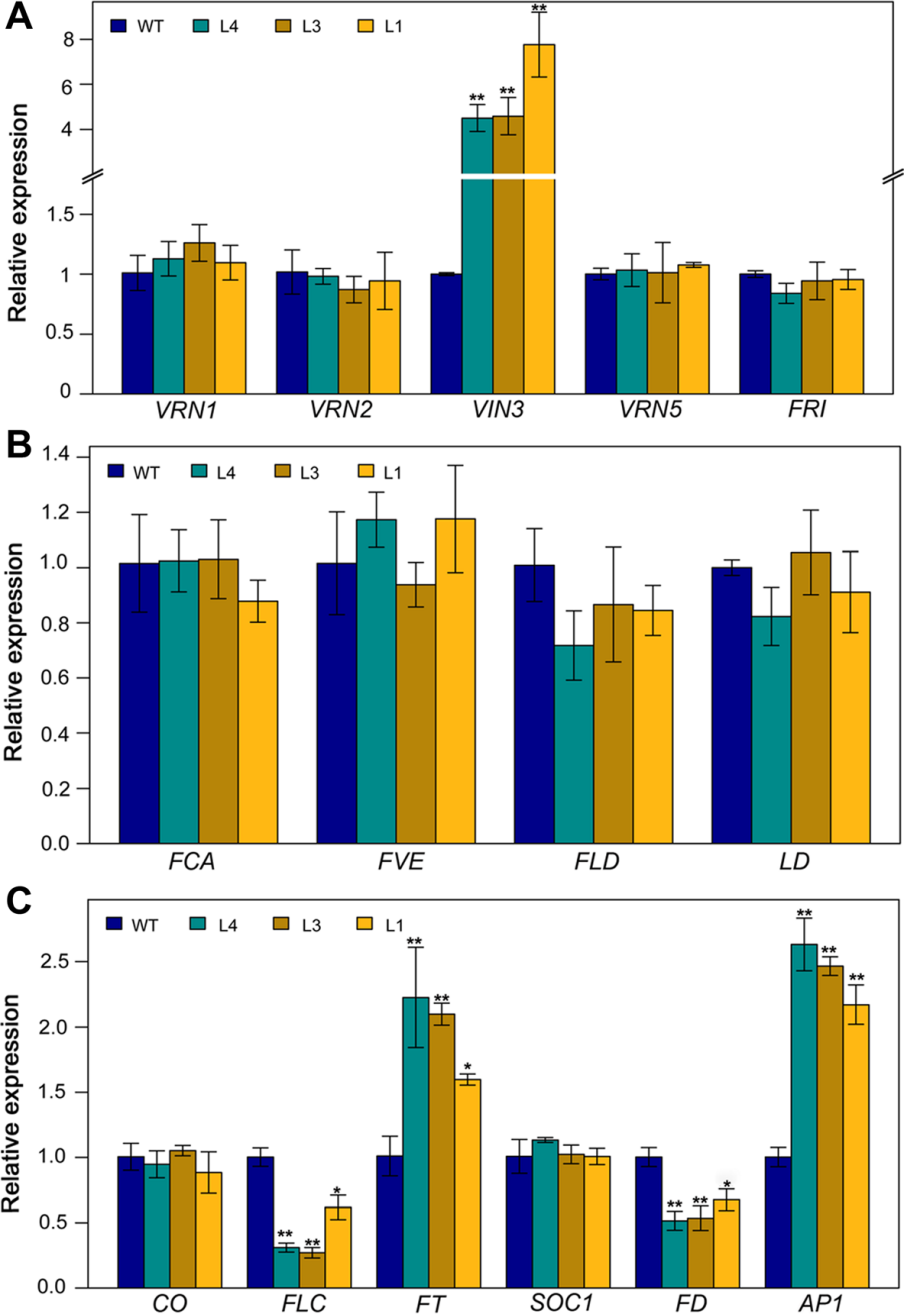
Quantitative real-time PCR analysis of several flowering-time genes in transgenic and wild-type (Col-0) plants. **a** Expression levels of vernalization pathway genes of Arabidopsis. **b** Expression levels of autonomous pathway genes of Arabidopsis. **c** Expression levels of other genes related to flowering time in Arabidopsis. Soybean (Glyma20g27280) and Arabidopsis (AT5G62690) β*-tubulin* were used as internal controls for normalization of soybean and Arabidopsis samples, respectively. Values are means ± SD of three biological replicates, with each measurement repeated three times. Significant differences according to the *t*-test are denoted as follows: * *p* < 0.05, ** *p* < 0.01. WT means wild-type Arabidopsis; L4, L3 and L1 refer to independent transgenic lines

We over-expressed *Glyma11g13220* in Arabidopsis (Col-0) to evaluate the function of this gene in regulation of flowering time. Three transgenic T_2_ lines with the most obvious flowering time phenotypes were chosen to assess the expressions of genes involved in flowering pathways. Notably, over-expression of *Glyma11g13220* resulted in obvious early flowering. The flowering times of transgenic Arabidopsis lines L4, L3 and L1 were respectively about 4, 4 and 3 days earlier than the wild type (Col-0) (Fig. 6a, d) and correlated with *Glyma11g13220* expression levels (Fig. 6a, b, d). Over-expression of *Glyma11g13220* also led to remarkable changes in rosette leaf numbers of L4 and L3 (Fig. 6c). To further confirm the possible pathway by which *Glyma11g13220* stimulated flowering, we evaluated the expressions of several genes involved in different flowering pathways. qRT-PCR analysis indicated that transcript levels of *FLC* and *FD* in transgenic Arabidopsis decreased significantly compared with the wild type (Col-0), whereas *VIN3, FT* and *AP1* noticeably increased (Fig. 7). To summarize, the early flowering of transgenic Arabidopsis may have been due to the decreased expression of the floral repressor *FLC*.

### Effects of low temperature treatment on *Glyma11g13220* expression

**Fig. 8:**
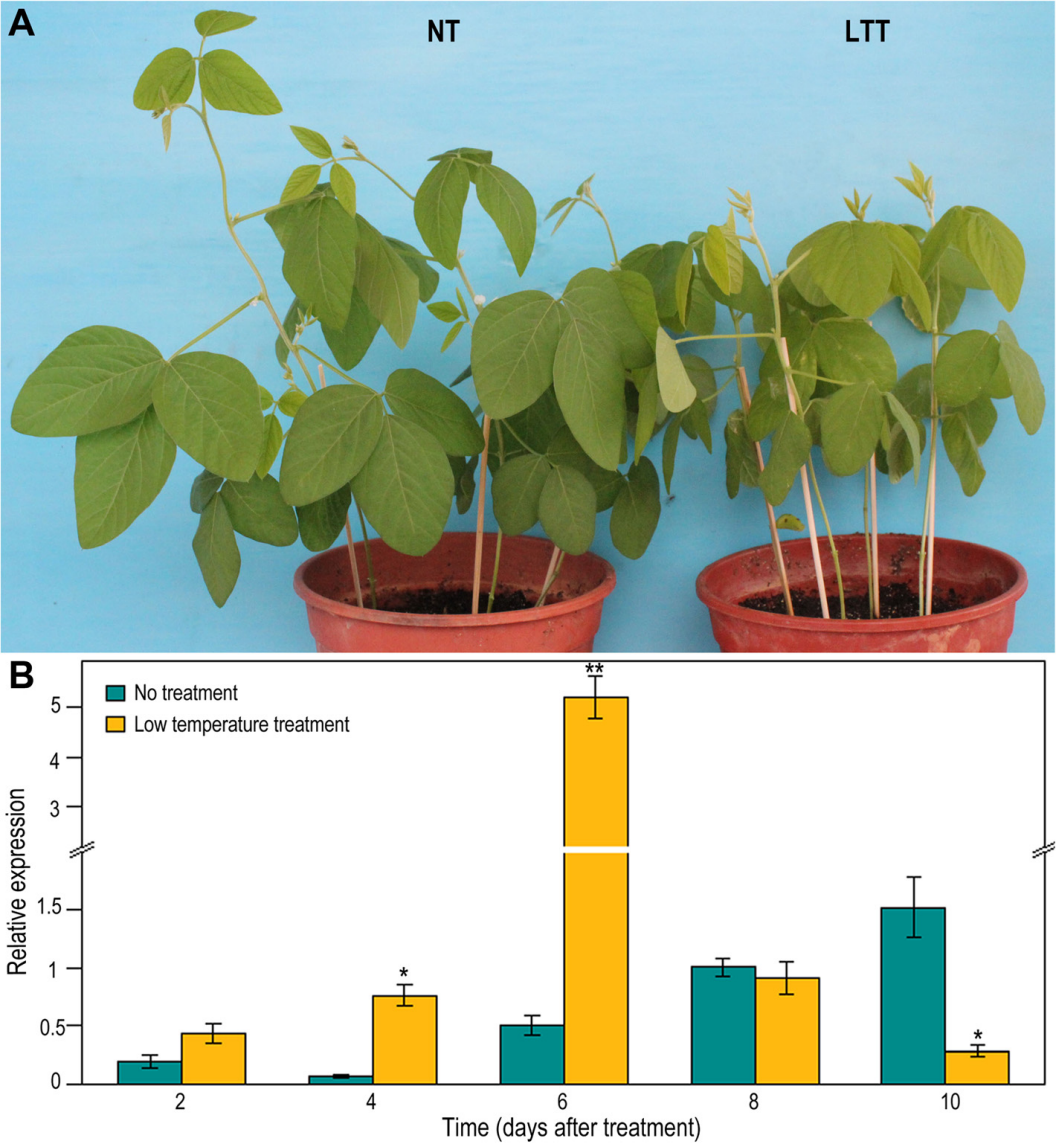
Effects of low temperature treatment on *Glyma11g13220* expression. **a** Soybean plants during initial flowering. NT, no treatment; LTT, low temperature treatment. **b** Quantitative real-time PCR analysis of *Glyma11g13220* under no- and low temperature treatments at 2, 4, 6, 8 and 10 days after treatment. Values are means ± SD of three biological replicates, with each measurement repeated three times. Significant differences according to the *t*-test are denoted as follows: * *p* < 0.05, ** *p* < 0.01

To investigate whether *Glyma11g13220* is affected by low temperature, soybean plants were exposed to a low temperature treatment (8 h at 15 °C/16 h at 13 °C day/night) for 10 days and then returned to normal temperature conditions. Compared with the flowering time of untreated plants, that of low-temperature-treated plants was delayed by approximately 8 days (Additional file 3). After 2, 4 or 6 days of treatment, *Glyma11g13220* expression in treated plants was up-regulated relative to untreated ones. By day 6 of treatment, *Glyma11g13220* expression was highly significantly different between treated and untreated plants. After treatment for 8 or 10 days, *Glyma11g13220* expression was decreased in treated plants compared with the untreated controls (Fig. 8). These results imply that *Glyma11g13220* can respond to low temperature and may play a role in low-temperature-induced delay of flowering of soybean.

## Discussion

Research on the regulation of flowering time has been carried out for more than a century [28]. Because it is sensitive to photoperiod, soybean is considered to be a typical photoperiodic model plant. Many researchers have consequently focused on soybean photoperiod pathway genes, which give rise to the identification of the functions of photoperiod pathway genes such as *GmFTs* and *GmCOs* [29–34]. Comparative genomic analysis of soybean flowering genes following the release of the draft cultivated soybean sequence has revealed that the soybean genome contains flowering regulation pathways similar to those of Arabidopsis [22, 23, 35]. Interestingly, the soybean genome has retained vernalization pathway genes over the course of evolution, even though flowering in soybean does not require prolonged exposure to low temperature [22]. Little is known, however, about the functions of these vernalization pathway genes in soybean and whether the pathway is redundant. In this study, we investigated the functions of *Glyma11g13220*, a homolog of Arabidopsis *VRN1*. Our generated data provide the first evidence to show that Glyma11g13220 is a functional protein that may regulate flowering time through the vernalization pathway in Arabidopsis. Our results also suggest that the preservation of vernalization pathway genes in soybean is meaningful and that *Glyma11g13220* may play an important role in low-temperature-induced delay of flowering of soybean. In addition, we found that the function of Arabidopsis *VRN1* and *Glyma11g13220* is both conserved and divergent.

Vernalization is the process in which plants are induced to flower after exposure to prolonged low temperature [12]. Recent studies have explored vernalization response at the molecular level in three plant families: Poaceae, Brassicaceae and Amaranthaceae [36]. Although designated by the same names, the genes related to vernalization response differ greatly in function among different plant families [36]. For example, wheat and barley *VRN1* genes encode MADS-box transcription factors [37], whereas the Arabidopsis *VRN1* gene contains two B3 DNA domains promoting flowering and is predicted to be involved in epigenetic repression of *FLC* [13, 38]. Previous studies have revealed the conserved nature of flowering pathways between soybean and Arabidopsis [33, 39, 40]. In our research on soybean, we also found that the vernalization pathway is apparently conserved between Arabidopsis and soybean. In Arabidopsis, *VRN1* encodes two B3 DNA domains and localizes in the nucleus [13]. Overexpression of *VRN1* causes early flowering and stably represses *FLC*, the major vernalization pathway gene target, in Arabidopsis [13]. Glyma11g13220 also encodes two B3 DNA domains and is nuclear-localized according to our study (Figs. 1 and 5). Over-expression of *Glyma11g13220* was found to result in early flowering in Arabidopsis (Col-0) (Fig. 6a, d). Furthermore, heterologous expression of *Glyma11g13220* caused down-regulation of *FLC*, a floral repressor, and significant up-regulation of *FT* in transgenic Arabidopsis (Fig. 7). These altered expressions should be responsible for the early flowering phenotype of transgenic Arabidopsis.

Functional divergence exists between Arabidopsis *VRN1* and *Glyma11g13220. VRN1* is constitutively expressed in Arabidopsis [13], while *Glyma11g13220* is mainly expressed in soybean leaves and pods (Fig. 3). Apart from this distinction, we found many light-responsive *cis*-elements in the *Glyma11g13220* promoter (Table 1), and our time course-dependent experiment demonstrated that *Glyma11g13220* can respond to photoperiod (Fig. 4). Over-expression of *VRN1* affected other phenotypes as well. *VRN1* over-expression down-regulated *FLC*, but only slightly, compared with the effect of *Glyma11g13220* over-expression in Arabidopsis. In addition, *FD* was down-regulated and *AP1* noticeably up-regulated in transgenic Arabidopsis (Fig. 7)*.* FD, a bZIP transcription factor, is highly expressed at the shoot apex, and its levels decrease soon after the floral primordium begins to express *AP1*. This transcription factor can also interact with FT protein at the shoot apex. A complex of FT and FD proteins activates floral identity genes such as *AP1* [41, 42]. *AP1* up-regulation, which marks a commitment to flower formation [43], was ultimately responsible for earlier flowering of transgenic plants compared with the wild type (Fig. 6a, d). Interestingly, *VIN3* expression was found to be significantly induced in transgenic Arabidopsis (Fig. 7). Previous studies have shown that *VIN3* is expressed only in Arabidopsis during vernalizing cold and contributes to the establishment of *FLC* repression during vernalization [18, 19]. In other words, *VIN3* expression is a marker of vernalization, with *FLC* repression not occurring until *VIN3* is induced [19]. In our transgenic lines, however, *VIN3* was significantly up-regulated without vernalization, implying that *Glyma11g13220* may be associated with low temperatures. Our subsequent experiment revealed that *Glyma11g13220* can respond to low temperature (Fig. 8). Consequently, we speculate that *Glyma11g13220* is photoperiod responsive at normal temperatures in soybean. *Glyma11g13220* may play a pivotal role in the regulation of flowering time when low temperatures are suddenly encountered, thereby ensuring reproductive success.

## Conclusions

The functional protein Glyma11g13220 may regulate flowering time through the vernalization pathway in Arabidopsis and can respond to photoperiod and low temperature in soybean. Although soybean does not need to be vernalized for flowering, the vernalization pathway gene of soybean is functional. Finally, *Glyma11g13220* and Arabidopsis *VRN1* have conserved as well as divergent functions.

## Methods

### Plant materials and growth conditions

Huachun5, a soybean cultivar bred by the Guangdong Subcenter of the National Center for Soybean Improvement, was used in this study. Soybean seedlings were grown in pots containing a 3:1 mixture of turf soil and vermiculite in a growth chamber at 28 °C. Day-length regimes consisted of either short-day (8-h light/16-h dark) or long-day (16-h light/8-h dark) conditions.

The Arabidopsis Col-0 ecotype was used as the wild type in this experiment. Seeds of Arabidopsis, both wild-type and transgenic lines, were surface sterilized, plated on half-strength Murashige and Skoog agar medium, and incubated in darkness for 2 days at 4 °C. The plates were then moved into a growth chamber maintained at 22 °C under long-day conditions without vernalization. Seven days later, seedlings were transplanted into pots containing 3:1 turf soil and vermiculite and grown under long-day conditions at 22 °C.

### Total RNA extraction and cDNA cloning of *Glyma11g13220*

Total RNA was extracted from plant samples using Trizol reagent (Invitrogen, USA) according to the manufacturer’s instructions. RNA quality was assessed with a NanoDrop 2000c spectrophotometer (Thermo Scientific, USA) at three difference absorbances: 230, 260 and 280 nm. RNA integrity was verified by 2 % agarose gel electrophoresis. One microgram of DNase-treated RNA was then subjected to reverse transcription using a PrimeScript RT Reagent kit with gDNA Eraser (Takara, Japan).

For isolation of *Glyma11g13220* cDNA, total RNA was extracted from soybean shoots at the fourth trifoliate stage. The full-length of *Glyma11g13220* was amplified using specific primers VRN1-F and VRN1-R (Additional file 4) from synthesized cDNA and subcloned into a pZeroBack/blunt vector (Tiangen, China) for sequencing.

### Bioinformatics analysis of *Glyma11g13220*

Homologous protein sequences of Glyma11g13220 were identified from NCBI and Phytozome v.9.1 databases [25, 44]. Amino acid sequence alignment was carried out using Clustal Omega [45]. A phylogenetic tree was constructed based on the aligned set of amino acid sequences according to the neighbor-joining algorithm in MEGA 5.0 software [46] with 1,000 bootstrap replicates. Information on the *Glyma11g13220* promoter sequence was retrieved from the Phytozome v.9.1 database [25]. The 1,500-bp sequence upstream of the *Glyma11g13220* start codon was designated as the promoter. *Cis*-acting elements in the *Glyma11g13220* promoter were analyzed using the PLANTCARE program [27].

### qRT-PCR analysis

qRT-PCR was performed on a CFX96 Real-Time PCR Detection System device (Bio-Rad, USA) using a SsoFast EvaGreen Supermix kit (Bio-Rad). All reactions were carried out in 20-μl volumes containing 1 μl cDNA as a template. Thermal cycling conditions consisted of 95 °C for 3 min, followed by 40 cycles of 95 °C for 10 s, 57.0–63.3 °C (depending on the gene) for 10 s and 72 °C for 30 s. β*-tubulin* genes of soybean (Glyma20g27280) and Arabidopsis (AT5G62690) were used as internal controls to normalize samples from those two species. Each PCR assay included three biological replicates and three technical replicates. The qRT-PCR data were evaluated by the 2^−ΔΔCt^ method [47]. The specific primers used for each gene are listed in Additional file 4.

### Expression analyses of *Glyma11g13220* in soybean

To study the expression pattern of *Glyma11g13220* in different organs at different soybean developmental stages, we collected plant organs, such as roots, steams, leaves, shoot apexes (including the apical meristem and immature leaves), flowers and pods, from three individual plants 12 h after dawn.

For time course-dependent expression analyses, all seedlings were grown under short-day conditions until 10 DAE, at which point half of the seedlings were transferred to long-day conditions. Fully expanded trifoliate leaves of three individual plants growing under short- and long-day conditions were sampled 12 h after dawn at 12, 15, 18, 21, 24, 27 and 30 DAE. All samples were immediately frozen in liquid nitrogen and stored at −80 °C until further processing.

### Subcellular localization of Glyma11g13220 protein

To generate a *35S::Glyma11g13220-eGFP* recombinant plasmid for the transient expression experiment, the full-length coding sequence of *Glyma11g13220* without a stop codon was amplified using primers 35SVRN1GFP-F and 35SVRN1GFP-R (Additional file 4). The resulting amplicon was digested with restriction enzymes BamHI and KpnI and inserted into a pYL322-d1-eGFP vector. The fusion vectors *35S::Glyma11g13220-eGFP* and empty control *35S::eGFP* were then used separately to co-transform rice leaf protoplasts with the construct *35S::ARF19IV-mCherry*, a nuclear localization marker [48, 49]. The eGFP and mCherry fluorescence signals from protoplasts were monitored with a confocal laser microscope (Carl Zeiss, OKO, Germany). At least 10 cells were examined in each sample.

### Ectopic expression of *Glyma11g13220* in Arabidopsis

The open reading frame of *Glyma11g13220* was amplified from the *pZeroBack-Glyma11g13220* vector using primers 35SVRN1-F and 35SVRN1-R (Additional file 4). The generated DNA fragment was cloned at BamHI and KpnI restriction sites into a pCAMBIA1301 binary vector driven by the cauliflower mosaic virus 35S promoter. This expression plasmid was transformed into *Agrobacterium tumefaciens* GV3101. Arabidopsis (Col-0) transformations were carried out using the floral dip method [50].

### Bioassays in *Glyma11g13220*-overexpressing Arabidopsis

Transgenic plant seeds were selected on half-strength Murashige and Skoog agar medium supplemented with 25 mg/L hygromycin. Transgenic seeds of each generation were harvested from individual seedlings. The T_2_ transgenic homozygous lines were chosen for further analyses, including phenotype characterization and determination of expression levels of *Glyma11g13220* and potential downstream genes (Additional file 3). Expression levels were detected by qRT-PCR.

### Low temperature treatment

Huachun5 seedlings were initially grown in a growth chamber under conditions of 8 h of daylight at 28 °C and 16 h of darkness at 26 °C. At the fourth trifoliate stage, half of the soybean plants were transferred to another growth chamber set to 8 h–15 °C/16 h–13 °C (day/night) and grown for 10 days (low temperature treatment). Leaves were sampled from three individual plants every 2 days. After completion of the low temperature treatment, the plants were returned to the growth chamber (8 h–28 °C/16 h–26 °C day/night) and flowering time was recorded. Untreated soybean plants were grown as controls in the growth chamber (8 h–28 °C/16 h–26 °C day/night), while the plants were treated to low temperature. Leaves were sampled from three individual control plants every 2 days at the same collection time used for the low temperature-treated plants.

### Data analysis

All data were represented as the mean ± SD of three biological replicates. Student’s *t*-test at *p* < 0.01 or *p* < 0.05 was used to identify differences between observations.

## Availability of supporting data

The coding DNA sequence and translated protein sequence of *Glyma11g13220* supporting the results of this article are available through NCBI’s GenBank under the accession number KT321660 (http://www.ncbi.nlm.nih.gov/genbank). The phylogenetic trees were deposited in treebase (http://treebase.org) under following URL: http://purl.org/phylo/treebase/phylows/study/TB2:S18010?x-access-code=3f9ef9c0b00b8994eaf24c28c847e82a&format=html.

## Additional files

Additional file 1:
**Phylogenetic analysis of putative** ***Glyma11g13220*** **homologs between soybean and Arabidopsis.** (TIFF 79319 kb) Additional file 2:
**Aligned amino acid sequences of Glyma11g13220 and Arabidopsis VRN1.** (TIFF 1978 kb) Additional file 3:
**Initial flowering dates of soybean plants.** SD and LD refer to initial flowering dates of soybean plants grown under short- and long-day conditions, respectively; LTT and NT respectively correspond to initial flowering dates of soybean plants subjected to low-temperature or control treatments; HC5, Huachun5. (PDF 85 kb) Additional file 4:
**Accession numbers and primers used in this study.** (DOCX 17 kb)

## Abbreviations

vrn1: Vernalization 1
vrn2: Vernalization 2
vin3: Vernalization insensitive 3
vrn5: Vernalization 5
co: Constans
flc: Flowering locus c
ft: Flowering locus t
soc1: Suppressor of constans 1
lfy: Leafy
ap1: Apetala 1
vp1: Viviparous 1
Col-0: Columbia-0
qRT-PCR: Quantitative real-time PCR
UTR: Untranslated region
DAE: Days after emergence
SD: Standard deviation
SD: Short day
LD: Long day
NT: No treatment
LTT: Low temperature treatment
