# Supplementary figures and images for: Glyma11g13220, a homolog of the vernalization pathway gene VERNALIZATION 1 from soybean [Glycine max (L.) Merr.], promotes flowering in Arabidopsis thaliana
Source: BMC Plant Biol. 2015 Sep 29;15:232. doi: 10.1186/s12870-015-0602-6 (PMC4588262; doi:10.1186/s12870-015-0602-6)

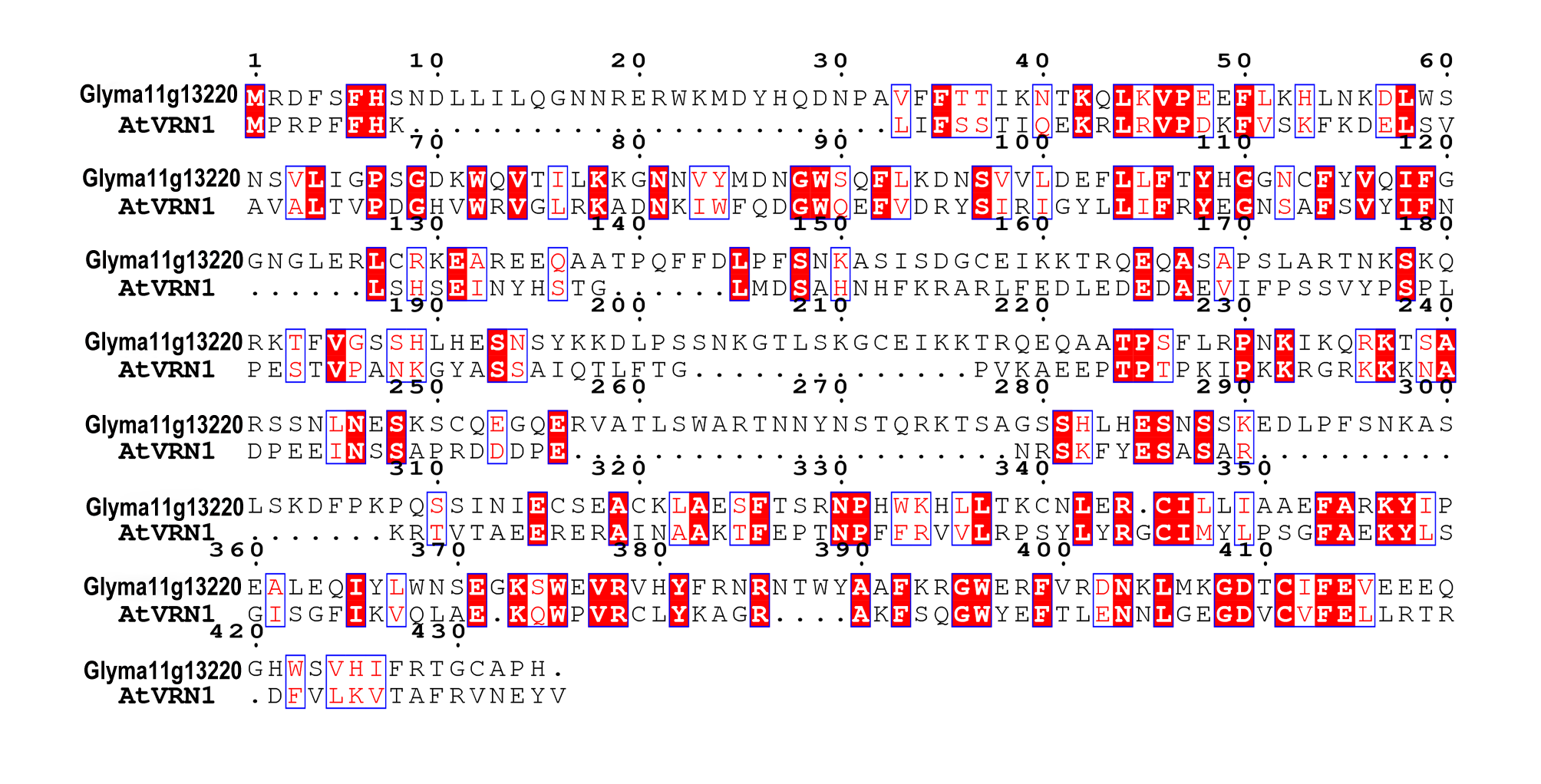

Supplement: Additional file 2: — Aligned amino acid sequences of Glyma11g13220 and Arabidopsis VRN1. (TIFF 1978 kb) [file 12870_2015_602_MOESM2_ESM.tiff]
