# Supplementary material for: Glyma11g13220, a homolog of the vernalization pathway gene VERNALIZATION 1 from soybean [Glycine max (L.) Merr.], promotes flowering in Arabidopsis thaliana
Source: BMC Plant Biol. 2015 Sep 29;15:232. doi: 10.1186/s12870-015-0602-6 (PMC4588262; doi:10.1186/s12870-015-0602-6)
Supplement: Additional file 3: — Initial flowering dates of soybean plants. SD and LD refer to initial flowering dates of soybean plants grown under short- and long-day conditions, respectively; LTT and NT respectively correspond to initial flowering dates of soybean plants subjected to low-temperature or control treatments; HC5, Huachun5. (PDF 85 kb) [file 12870_2015_602_MOESM3_ESM.pdf]

Initial flowering dates of soybean plants

	SD	LD	NT	LTT
HC5	29.33±1.97	53.67±0.75**	29.67±1.70	38.83±1.21**
