# Supplementary material for: Glyma11g13220, a homolog of the vernalization pathway gene VERNALIZATION 1 from soybean [Glycine max (L.) Merr.], promotes flowering in Arabidopsis thaliana
Source: BMC Plant Biol. 2015 Sep 29;15:232. doi: 10.1186/s12870-015-0602-6 (PMC4588262; doi:10.1186/s12870-015-0602-6)
Supplement: Additional file 4: — Accession numbers and primers used in this study. (DOCX 17 kb) [file 12870_2015_602_MOESM4_ESM.docx]

**Accesion numbers in this article.**

Sequence data from this article can be found in the public databases (GenBank/EMBL/Phytozome v.9.1) under the following accession numbers:

*SOC1,Arabidopsis,* AT2G45660; *FLC,Arabidopsis,*AT5G10140; *FVE,Arabidopsis*,AT2G19520; *FCA,Arabidopsis,*AT4G16280; *LD,Arabidopsis,*AT4G02560; *FLD,*

*Arabidopsis,*AT3G10390; *FT,Arabidopsis,*AT1G65480;*CO,Arabidopsis,*AT5G15840;*AP1,Arabidopsis,*AT1G69120; *VIN3,Arabidopsis,*AT5G57380; *VRN1,Arabidopsis,*AT3G18990; *VRN2,Arabidopsis,*AT4G1684; *FRI,Arabidopsis,*AT4G00650;

*TUB2,Arabidopsis,*AT5G62690; *VRN5,Arabidopsis,*AT3G24440; *Glycine max,* Glyma11g13220; *GmFLC,Glycine max,*Glyma05g28130; *Gm-β-tublin,Glycine max,*Glyma20g27280.

**The primers used in the experiment**

| **Accession No.** | **Primer set** | |
| --- | --- | --- |
|  | **Forward primer(5'-3')** | **Reverse primer(5'-3')** |
| **For gene isolation** | | |
| 35SVRN1 | CGCGGATCC(*BamHI*)ATGAGAGACTTTTCATTTCAT | CGGGGTACC(*KpnI*)CTAATGTGGTGCAC |
|  |  |  |
| **For vector construction of overexpression in *Arabidopsis*** | | |
| 35SVRN1 | CGCGGATCC(*BamHI*)ATGAGAGACTTTTCATTTCAT | CGGGGTACC(*KpnI*)CTAATGTGGTGCAC |
|  |  |  |
| **For vector construction of subbcellular localization** | | |
| 35SVRN1-GFP | CGGGGTACC(*KpnI*)ATGAGAGACTTTTCATTTCATT | CGGGATCC(*BamHI*)TATGTGGTGCACATCC |
|  |  |  |
| **For vector construction of yeast two-hybrid assay** | | |
| VRN1-PGBKT7 | CATGCCATGG(*NcoI*)GAATGAGAGACTTTTCATTTC | CGGGATCC(*BamHI*)CTAATGTGGTGCACATC |
|  |  |  |
| FLC-PGADT7 | AAAGAATTC(*EcoRI*)ATGGGGAAGAAGAAGCTG | CGCGGATCC(*BamHI*)CTATTTATTTATACTGAGTTC |
|  |  |  |
| **For qRT-PCR analysis** | | |
| Gm-*VRN1* | TGCTTATTGCTGCTGAGTTTGC | TCACGAACGAATCTTTCCCATC |
| Gm-*FLC* | TGACGCATAATCTGCTCCCTG | GCTAAACCATGGCATAGTTCCCT |
| Gm-*β-Tublin* | CCTCGTTCGAATTCGCTTTTTG | CAACTGTCTTGTCGCTTGGCAT |
| At-*VRN1* | GTTACTCCATTCGCATTGGTTATC | TGTGAGCGGAATCCATGAGAC |
| At-*VRN2* | AGCGTAGACAAAGAGGTGGCA | GGCTATGGCTGGTGGAATG |
| At-*VIN3* | TCTGTCTCAGAGTGGTTTCC | CAGTGTTCAGTGTTGTCCTT |
| At-*VRN5* | AGCACGAATCAAATGGGAAAC | GACACGATAAGCCACAGAACTCA |
| At-*FRI* | ACATATACGCGAATATCTCTG | TCTTCACCTTCCCTTTACCAC |
| At-*FCA* | TGGTCTAACGGGTGAAAGCAAGTG | TGCTGTTTCTGTTGCTCTCGTTC |
| At-*FLD* | TAACTCTAAGCGAGCCTCGTAAG | TCTGTTGCTGCTGGTTGAAAT |
| At-*LD* | CGAGTTATTAGCTGCGTTAC | ATTAGTTGATGGAGTGGGAG |
| At-*FVE* | AGCTGCTGTTCTTTGTGTTCAGTG | GCTGCACGATCAGACTTCTTACTG |
| At-*FLC* | GAGAATAATCATCATGTGGGAGC | CAACCGCCGATTTAAGGTGG |
| At-*CO* | CACTACAACGACAATGGTTCC | GGTCAGGTTGTTGCTCTACTG |
| At-*FT* | CCCTGCTACAACTGGAACAAC | AAGAACAAGGTAACCCAATGAAC |
| At-*SOCI* | AAACGAGAAGCTCTCTGAAAAG | AAGAACAAGGTAACCCAATGAAC |
| At-*AP1* | GCAAGCAATGAGCCCTAAAG | ACTGCTCCTGTTGAGCCCTA |
| At-*TUB2* | ATCGATTCCGTTCTCGATGT | ATCCAGTTCCTCCTCCCAAC |
